# Evaluation of monkey intraocular pressure by rebound tonometer

**Published:** 2009-10-27

**Authors:** Wenhan Yu, Guiqun Cao, Jinghua Qiu, Xuyang Liu, Jia Ma, Ni Li, Man Yu, Naihong Yan, Lei Chen, Iok-Hou Pang

**Affiliations:** 1 Ophthalmic Laboratories & Department of Ophthalmology, West China Hospital, Sichuan University, Chengdu, China; 2 Glaucoma Research, Alcon Research Ltd., Fort Worth, TX

## Abstract

**Purpose:**

To evaluate the usefulness of the TonoVet™ rebound tonometer in measuring intraocular pressure (IOP) of monkeys.

**Methods:**

The accuracy of the TonoVet™ rebound tonometer was determined in cannulated eyes of anesthetized rhesus monkeys where IOP was controlled by adjusting the height of a connected perfusate reservoir. To assess the applicability of the equipment through in vivo studies, the diurnal fluctuation of IOP and effects of IOP-lowering compounds were evaluated in monkeys.

**Results:**

IOP readings generated by the TonoVet™ tonometer correlated very well with the actual pressure in the cannulated monkey eye. The linear correlation had a slope of 0.922±0.014 (mean±SEM, n=4), a y-intercept of 3.04±0.61, and a correlation coefficient of r^2^=0.97. Using this method, diurnal IOP fluctuation of the rhesus monkey was demonstrated. The tonometer was also able to detect IOP changes induced by pharmacologically active compounds. A single topical ocular instillation (15 μg) of the rho kinase inhibitor, H1152, produced a 5–6 mmHg reduction (p<0.001) in IOP, lasting at least 4 h. In addition, topical administration of Travatan®, a prostaglandin agonist, induced a small transient IOP increase (1.1 mmHg versus vehicle control; p=0.26) at 2 h after treatment followed by a pressure reduction at 23 h (−2.4 mmHg; p<0.05). Multiple daily dosing with the drug produced a persistent IOP-lowering effect. Three consecutive days of Travatan treatment produced ocular hypotension of −2.0 to −2.2 mmHg (p<0.05) the following day.

**Conclusions:**

The rebound tonometer was easy to use and accurately measured IOP in the rhesus monkey eye.

## Introduction

Glaucoma leads to visual impairment and blindness in millions of people each year [1]. Elevated intraocular pressure (IOP) is a major risk factor of this disease. Unfortunately, the pathogenic mechanism of the disease is still unclear. Many animal models have been developed to study glaucoma at the molecular, cellular, and physiologic levels as well as to evaluate potential new treatments [2,3]. Among the various in vivo models, non-human primates are of great value because of their morphological and physiologic similarities to humans [4]. Practically all clinically approved IOP-lowering medications have been shown to lower IOP in the monkey [5]. Development of novel IOP-lowering therapies also often depends on critical preclinical findings provided by the monkey model. Therefore, an accurate, reproducible, and convenient IOP assessment for the monkey is central to the continuous success of glaucoma research. Equipments such as the Goldmann applanation tonometer, pneumatonometer, and the TonoPen™ applanation tonometer are commonly used for the purpose of measuring monkey IOP. However, some such as the Goldmann tonometer and pneumatonometer, albeit accurate and popular, are cumbersome to use. In contrast, the TonoPen™, although easy to use, was limited by its accuracy [6,7]. There is clearly an unmet need for an improved tonometer with excellent accuracy and convenience as well usefulness for non-human primate IOP appraisal.

More than a decade ago, a novel rebound tonometer (originally called induction/impact tonometer) was introduced [8]. To measure IOP, a light weight magnetic probe is aimed at and normal (perpendicular) to the center of the cornea. When triggered, it is propelled by a solenoid toward the cornea, impacts it, and rebounds. The deceleration of the probe during the impact, which correlates with IOP, can be monitored by the voltage change induced by the moving magnetic probe. It was demonstrated that IOP values in 71 eyes of 36 patients obtained by this method compare well with those obtained by the Goldmann tonometer [8]. Recently, two hand-held apparatuses based on this technical principle became commercially available for animal studies, the TonoLab™ rebound tonometer intended for use in rodents and the TonoVet™ tonometer for use in animals with bigger eyes. Rodent IOP readings obtained by the TonoLab™ have been reported to represent the actual IOP accurately and reproducibly [9,10]. The equipment was also reported to be easy to use with minimal required training [9,10].

In the present study, we evaluated the accuracy of the TonoVet™ tonometer for measuring IOP in rhesus monkeys. We compared IOP readings reported by the equipment with actual IOP preset by anterior chamber cannulation, similar to that performed in the rodents [9]. We also used the TonoVet™ to measure normal diurnal IOP fluctuation of sedated monkeys. Furthermore, we used the tonometer to monitor IOP levels following ocular topical treatment with IOP-lowering compounds, the rho kinase inhibitor H1152 and Travatan® ophthalmic solution, an ocular hypotensive medication approved for use in humans. Our results demonstrated the usefulness of the TonoVet™ for measuring IOP in rhesus monkeys.

## Methods

### Animals

Five male and five female rhesus monkeys (*Macaca mulatta*), two to six years of age, were obtained from the PingAn Monkey Breeding Base (Chengdu, China) through the National Chengdu Center for Safety Evaluation of Drugs, which is accredited by the Association for Assessment and Accreditation of Laboratory Animal Care International (AAALAC International). All animal experiments were conducted in compliance with the ARVO Statement for the Use of Animals in Ophthalmic and Vision Research, the Guide for the Care and Use of Laboratory Animals (National Research Council), and under the supervision of the Institutional Animal Care and Use Committee. One male and one female (M1 and M2; Table 1) were used to validate the accuracy of the tonometer. The remaining eight monkeys (M3–M10; Table 1) were subjected to diurnal IOP measurements and drug effects on IOP. The animals were housed under 12-h/12-h light/dark cycle with lights on starting at 6:00 AM. Food and water were available ad libitum. Physical examinations including an ophthalmic examination were conducted in all monkeys before the experiments to exclude potential health factors that might affect IOP measurements. Details pertaining to each monkey are described in Table 1.

**Table 1:** Information of monkeys used in the study.

**ID #**	**Age (years)**	**Sex**	**Weight (kg)**	**Studies**
M1	6	F	8.2	Tonometer validation
M2	6	M	7.8	Tonometer validation
M3	5	M	5	Diurnal and Drug Effects
M4	3	M	4.2	Diurnal and Drug Effects
M5	5	M	5.2	Diurnal and Drug Effects
M6	3	M	5.8	Diurnal and Drug Effects
M7	2	F	4.2	Diurnal and Drug Effects
M8	3	F	3.8	Diurnal and Drug Effects
M9	5	F	5.2	Diurnal and Drug Effects
M10	2	F	4.2	Drug Effects

### Rebound tonometer

IOP was measured using the TonoVet™ rebound tonometer (Tiolat Oy, Helsinki, Finland) according to the manufacturer’s recommended procedures. The equipment is programmed to average the IOP values of six consecutive, acceptable measurements and produce a reading of the mean IOP. In the following studies, five readings (each a mean of six measurements; a total of 30 separate measurements) were obtained under each condition. The tonometer was validated as described below before it was used to assess IOP in subsequent studies.

### Tonometer validation

Monkeys M1 (six-year-old female) and M2 (six-year-old male) were anesthetized by intravenous injection of pentobarbital sodium (40 mg/kg; Sigma-Aldrich China, Shanghai, China). With the animals lying on their sides, anterior chamber cannulation was performed consecutively on both eyes by inserting a 30-gauge needle through the cornea into the anterior chamber. The needle was connected via polyethylene tubing to a three-way connector, which was connected in parallel to a pressure transducer assembly (Biomechanical Engineering Laboratory, Sichuan University, Chengdu, China) and a fluid reservoir. The pressure transducer assembly, reservoir, and tubing were filled with Balanced Salt solution (BSS)® intraocular irrigating solution (Alcon Laboratories, Fort Worth, TX) before performing cannulation. Following cannulation, the height of the reservoir relative to the eye was adjusted to produce various pressure values (10, 15, 20, 25, 30, 35, and 40 mmHg, in random order) inside the eye. A researcher who was masked to the height of the reservoir and the pressure transducer readings used the TonoVet™ tonometer to obtain pressure readings. Five readings were made at each preset pressure for each eye.

### Measurement of monkey IOP

**Figure 1:**
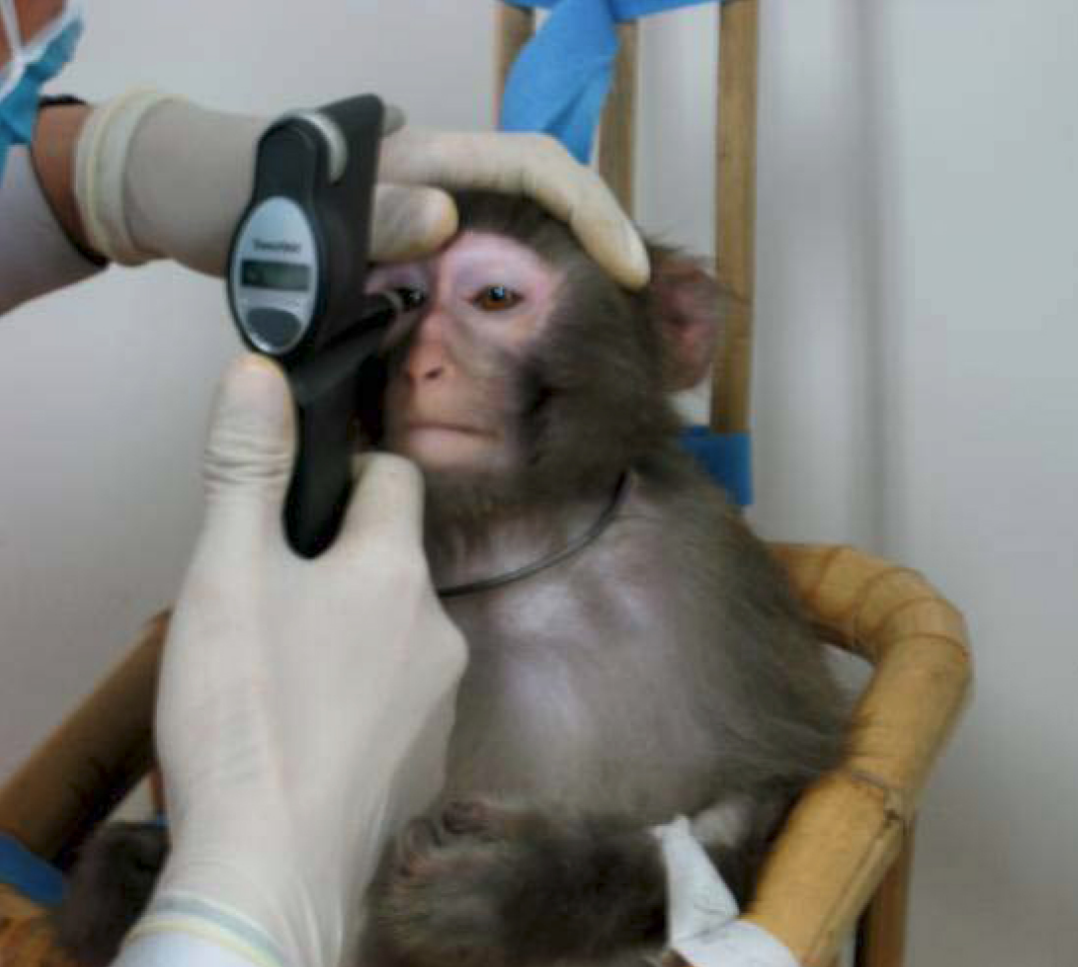
IOP measurement using TonoVet™. Each monkey was positioned in a custom-designed monkey chair with arms gently restrained, and IOP was measured with the TonoVet™ tonometer according to the manufacturer’s recommended procedures.

Eight (four male and four female) naïve rhesus monkeys (M3–M10) were used for IOP studies. For IOP measurements, sedation was induced by an intramuscular injection of ketamine (8 mg/kg; Jiangsu Hengrui Medicine Co. Ltd., Jiangsu, China). Each animal was positioned in a seated posture in a custom-designed monkey chair with arms gently restrained, and IOP for both eyes was measured (Figure 1). Five readings were obtained per eye for each condition, and the mean IOP value was calculated and reported.

### Drug effects on IOP

H1152 (Alexis Corp., Lausen, Switzerland) was dissolved in BSS to a final concentration of 0.05% (w/v). One drop (30 μl) of the solution was administrated topically to the right eye of six animals whereas the vehicle was administrated to the left eye. Assessments of the IOP were performed at 0 h (before drug treatment) as well as 2 h, 4 h, and 6 h post-treatment.

Travatan® ophthalmic solution (Alcon) or Eyestream® ocular irrigation solution (Alcon), which served as a control, was instilled ocular topically in a masked fashion to assigned monkey eyes. Half of the animals (two males and two females) were treated with 1 drop (30 μl) of Travatan® (0.004% travoprost) on their right eye and the remaining half was treated on their left eye. The contralateral eye received 1 drop (30 μl) of Eyestream®. In the first study, a single treatment was administered at 10:00 AM (0 h) immediately after a baseline IOP measurement. Post-treatment IOP was assessed at 2 h, 7 h, 23 h, and 47 h. In the second study, both Travatan® and Eyestream® (both 30 μl) were administered daily at 10:00 AM for three consecutive days (0 h, 24 h, and 48 h), and IOPs were measured at 0 h (immediately before the 0 h dosing), 24 h (immediately before the 24 h dosing), 48 h (immediately before the 48 h dosing), 72 h, and 96 h.

### Data analysis

Linear regression was used to evaluate linear correlation between the measured and actual IOP values in the tonometer validation studies. Comparison of the IOP values at various time points in the diurnal IOP fluctuation study was performed by one-way ANOVA. Two-tailed paired Student’s *t*-test was used to compare IOPs between drug- and vehicle-treated eyes. Data are reported as mean±standard error of mean (SEM). Differences are regarded as significant when p<0.05.

## Results

### Tonometer validation

**Figure 2:**
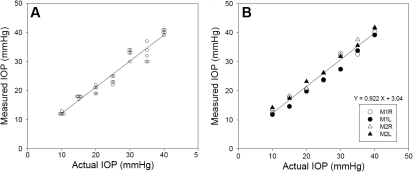
Correlation between IOP readings obtained by the TonoVet™ tonometer and the actual IOP values. Eyes of two anesthetized monkeys were cannulated and perfused with BSS. Their IOPs were manipulated by changing the height of the perfusate reservoir. TonoVet™ readings were obtained by a researcher unaware of the actual IOP. **A**: Examples of individual IOP readings of a randomly selected eye (Eye number: M1R; right eye of monkey M1). Each symbol represents a single reading. **B**: Mean IOP values of TonoVet™ tonometer measurements for each of the four tested monkey eyes. M1R and M1L represent the right and left eye, respectively, of monkey M1. M2R and M2L represent the right and left eye, respectively, of monkey M2.

The TonoVet™ rebound tonometer generated IOP readings that correlated very well in a linear relationship with the preset IOP values, which were confirmed by the pressure transducer assembly. An example of the tonometer readings at each preset IOP is shown in Figure 2A. The correlation was reproducible in independent measurements from three additional monkey eyes, each with a regression coefficient (r^2^) between 0.968 and 0.996 (Figure 2B). The slopes and y-intercepts of the regression lines were 0.922±0.014 (mean±SEM, n=4) and 3.04±0.61 mmHg, respectively. The correlation coefficient of the combined data is r^2^=0.97. Importantly, although the values obtained by the equipment had good correlation with the actual IOP, systematic errors were evidenced. The mean slope and y-intercepts were statistically significantly different from 1 and 0 (both p<0.01), respectively. To adjust for the minor systematic error, all subsequent IOP measurements were corrected according to the following equation: actual IOP=(Measured IOP–3.04 mmHg)/0.922.

### Diurnal IOP fluctuation

**Figure 3:**
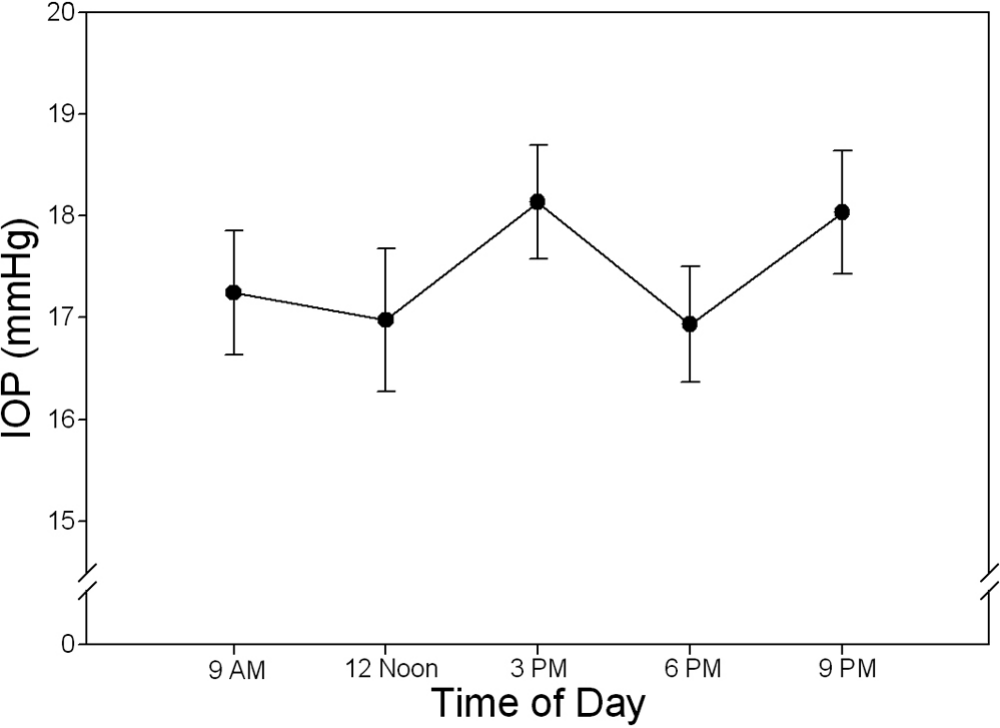
Normal diurnal IOP fluctuation in the rhesus monkey. Ketamine-treated animals were subjected to IOP measurement by the TonoVet™ tonometer at the indicated times of the day. Symbols represent mean±SEM (n=14).

A trend of diurnal IOP fluctuation of the rhesus monkey was detectable using the rebound tonometer. Measurements of IOP were conducted at 9:00 AM, noon, 3:00 PM, 6:00 PM (immediately before lights off), and 9:00 PM in the same day. For the 9:00 PM measurement, sedation and IOP measurement were performed under very dim light. The lowest IOP values were recorded at noon and 6:00 PM (17.0±0.7 mmHg and 16.9±0.6 mmHg, respectively; mean±SEM, n=14 eyes of seven monkeys). The IOP peaked at 3:00 PM (18.1±0.6 mmHg) and 9:00 PM (18.0±0.6 mmHg; Figure 3). However, the differences in IOPs at the various time points were not statistically significant (p>0.05 by one-way ANOVA).

### Effect of H1152 on monkey IOP

**Figure 4:**
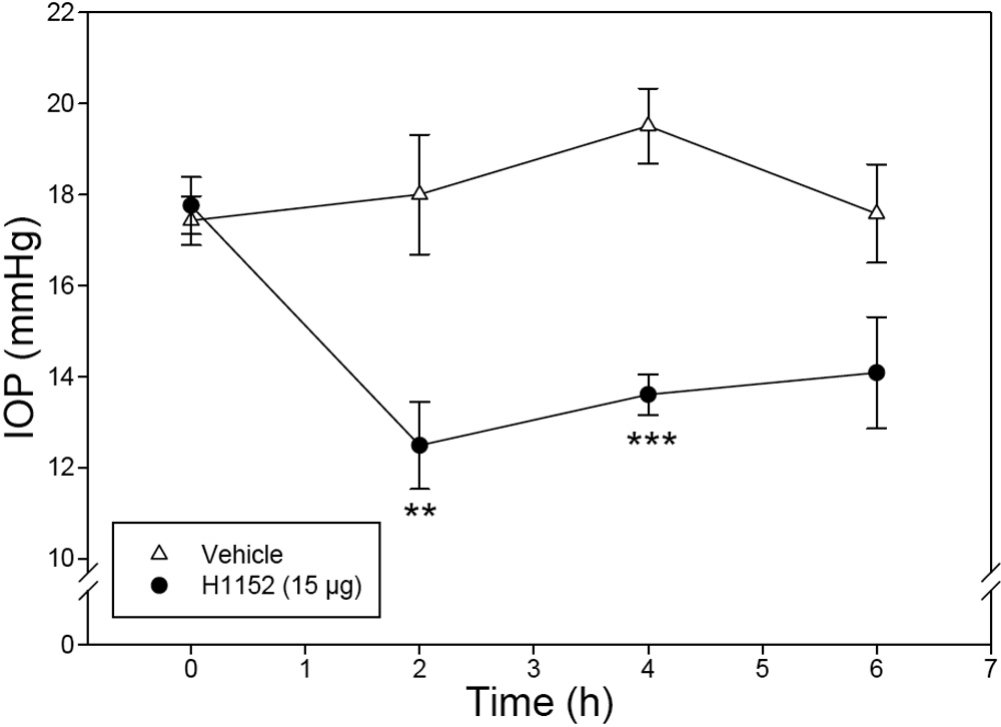
Effect of H1152 (15 μg) on monkey IOP. H1152 or vehicle was administered topically on assigned eyes at 0 h. Assessment of IOP was conducted by a researcher who was unaware of the treatments at 0 h immediately before dosing and at 2 h, 4 h, and 6 h post-treatment. Error bars represent SEM of IOP values (n=6). The double asterisk indicates p<0.01 and three asterisks denote p<0.001 between the two groups by paired Student’s *t*-test.

To determine if the TonoVet™ tonometer is useful in assessing IOP changes induced by pharmacologically active compounds, eyes of six monkeys were treated with H1152, a well known rho kinase inhibitor and ocular hypotensive. As shown in Figure 4, topical ocular administration of H1152 (15 μg, 30 μl of a 0.05% solution) produced a profound reduction in IOP compared to the vehicle-treated eyes: 5.5 mmHg (p<0.01) at 2 h, 5.9 mmHg (p<0.001) at 4 h, and 3.5 mmHg (p>0.05) at 6 h. These findings indicate that the instrument was effective in detecting drug-induced IOP changes in the monkey eye.

### Effects of Travatan® on monkey IOP

**Figure 5:**
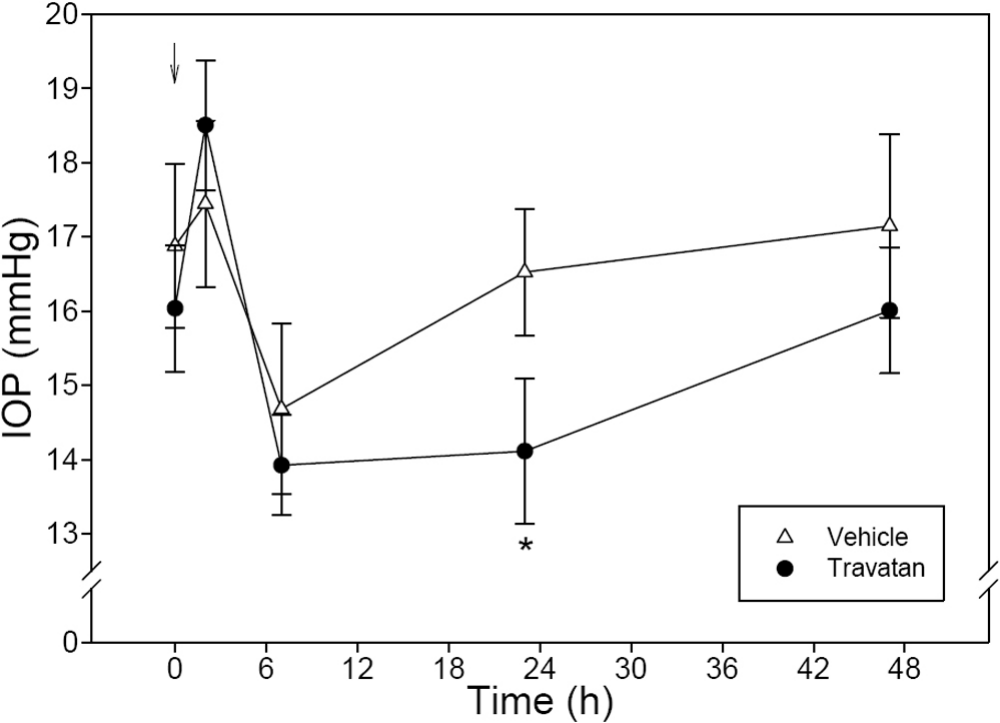
Effect of a single dose of 30 μl Travatan® on monkey IOP. Travatan® or Eyestream® (vehicle) was administered topically on assigned eyes at 0 h (10:00 AM). Assessment of IOP was conducted by a researcher who was unaware of the treatments at 0 h immediately before dosing and 2 h, 7 h, 23 h, and 47 h post-treatment. The arrow indicates when the treatment was applied. Error bars represent SEM of IOP values (n=8). An asterisk indicates p<0.05 between the two groups by paired Student’s *t*-test.

The TonoVet™ tonometer was also useful in revealing the IOP-lowering effect of Travatan®, a widely used ocular hypotensive drug that is approved for use in humans. Administration of a single drop (30 μl) of Travatan® onto the eye produced a transient but not statistically significant (p=0.26) IOP increase (1.1 mmHg) 2 h after treatment in monkeys under sedation. This tendency of ocular hypertension disappeared at 7 h. At 23 h, the Travatan®-treated eyes had a significantly (p<0.05) lowered IOP (−2.4 mmHg) compared to the placebo-treated eyes. The IOP reduction produced by Travatan® became insignificant 48 h after treatment (Figure 5).

**Figure 6:**
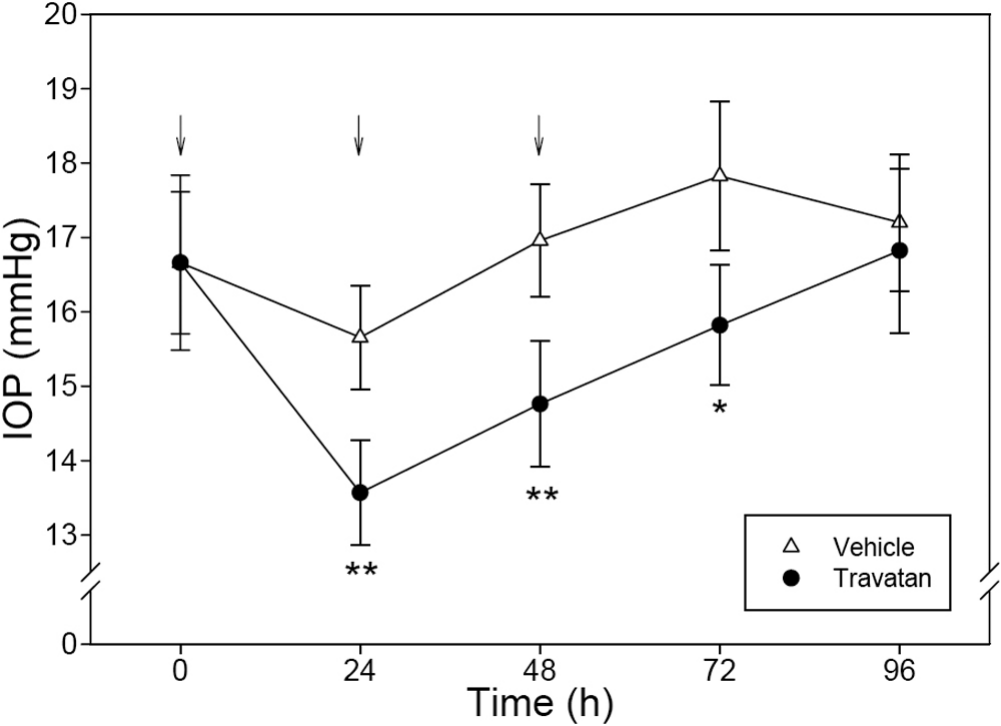
Effect of multiple doses of Travatan® on monkey IOP. One drop (30 μl) of Travatan® or Eyestream® (placebo) was administered topically on assigned eyes at 0 h (10:00 AM), 24 h, and 48 h (indicated by the arrows). Assessment of IOP was conducted by a masked researcher who was unaware of the treatments at 0 h immediately before dosing, 24 h immediately before dosing, 48 h immediately before dosing, 72 h, and 96 h post-treatment. Error bars represent SEM of IOP values (n=8). An asterisk denotes p<0.05 and a double asterisk denotes p<0.01 between the two groups by paired Student’s *t*-test.

These findings were confirmed by a second study in which daily treatments with Travatan® were administered in three consecutive days. As demonstrated in Figure 5, the rebound tonometer recorded a significant (p<0.05 or p<0.01) reduction in IOP (ranging from −2.0 mmHg to −2.2 mmHg) 24 h after each treatment. The drug effect vanished 48 h after the last drug treatment (Figure 6).

## Discussion

In this study, we reported the characterization and validation of the TonoVet™ rebound tonometer, a newly developed tonometer, in measuring IOP in rhesus monkeys. In the validation studies, using procedures recommended by the manufacturer, we observed linear correlations with very high correlation coefficients between the measured IOP values and the actual pressure within the eye. We further found that the measured IOP tended to overestimate slightly at lower actual IOP values and underestimate at higher IOP values such that the slope of the regression line was smaller than 1 while the y-intercept was larger than 0. To compensate for this systematic error and calculate the actual IOP, correction factors were needed.

With this equipment, we could detect diurnal IOP fluctuations in the sedated rhesus monkeys. In this study, IOP was lower at noon and 6:00 PM and higher at 3:00 PM and 9:00 PM with a 1.2 mmHg difference between them. Our findings of a higher IOP in the afternoon agree with published reports. For example, Bito et al. [11] showed that the IOP of free-breeding young rhesus monkeys peaked between 2–3 PM. Similarly, the IOP of cynomolgus monkeys was also higher between 2:00 PM and 4:00 PM [12]. In addition, our observations of lower IOP at noon and 6:00 PM corroborate with these previous reports. However, these investigators also observed an additional high level of IOP in the morning [11,12], which we did not detect. Furthermore, the current trend of diurnal fluctuation had only a small difference (1.2 mmHg) between the highest and lowest IOP values, and no statistical significance was reached among the various time points. These disparities may be related to sedation during IOP measurement. To address this issue, we are training monkeys to accept TonoVet™ measurements under conscious state without sedation.

We evaluated the practicability of using the tonometer to assess drug-induced IOP changes in the monkey. Our results indicated that the TonoVet™ was sensitive to detect the effects of IOP-lowering drugs. For example, inhibitors of rho kinase (also known as rho-associated coiled coil-forming kinase or ROCK) were shown to reduce rabbit and monkey IOP efficaciously by improving outflow facility [13-15]. In the current study, the TonoVet™ demonstrated the IOP-lowering effect of a prototypical rho kinase inhibitor, H1152, in the rhesus monkey. The drug produced more than a 5-mmHg decrease in IOP, which lasted for at least 4 h.

The TonoVet™ was further tested for its detection of IOP change generated by Travatan®, which contains 0.004% travoprost. Travoprost is the prodrug of a potent FP prostaglandin receptor agonist [16]. FP prostanoids such as travoprost and latanoprost are potent and efficacious IOP-lowering drugs in humans and other animals [16,17]. Interestingly, prostanoids typically cause a reduction of primate IOP including human with a 2–4 h lag time after topical administration, and the effect lasted for at least 24 h [18-21]. In the current study, we confirmed the prolonged IOP-lowering effect of travoprost. However, we unexpectedly observed a transient though not statistically significant increase of IOP 2 h after drug administration. To our knowledge, this initial, transient ocular hypertension was not previously reported in the rhesus monkey. Nonetheless, this phenomenon has been observed in the rat [22,23] and mouse [24]. The mechanism of action of the ocular hypertensive effect is unclear. And the discrepancies between these data are yet to be reconciled, although the differences in basal IOP levels and the use of sedative likely contributed to the intriguing observations.

In summary, we found that monkey IOP readings reported by the TonoVet™ rebound tonometer correlated well with the actual IOP values. Only small correction factors were needed to accurately estimate the actual IOP. The equipment is a very practical tool for monkey IOP measurement. It is easy to learn and convenient to use. It is non-invasive and well tolerated by the animal. We showed in this study that up to five measurements in one day and up to five consecutive daily measurements were tolerable to the animal. These repeated TonoVet™ readings did not cause any ocular abnormalities. We also showed that the instrument could detect diurnal IOP fluctuations commonly seen in mammals such as the rhesus monkey. Moreover, we conducted masked experiments to evaluate the feasibility of using the tonometer to monitor drug-induced IOP change in the monkey. Our results indicated that the TonoVet™ was able to discern the IOP-lowering effects of H1152 and Travatan. Overall, our data demonstrated the accuracy and applicability of this tonometer for IOP measurement in rhesus monkeys.
